# Radiosterilized Pig Skin, Silver Nanoparticles and Skin Cells as an Integral Dressing Treatment for Burns: Development, Pre-Clinical and Clinical Pilot Study

**DOI:** 10.3390/pharmaceutics15082105

**Published:** 2023-08-09

**Authors:** Carmina Ortega-Sánchez, Mario Pérez-Díaz, Yaaziel Melgarejo-Ramírez, Mario Chopin-Doroteo, Phaedra Silva-Bermudez, Valentín Martínez-López, Noé Zacaula-Juárez, Yessica Zamudio-Cuevas, Carmen Hernández-Valencia, Luis Esaú López-Jácome, Alberto Carlos-Martínez, Naxieli Reyes-Medina, Luis Tamez-Pedroza, María Esther Martínez-Pardo, María de Lourdes Reyes-Frías, Hugo Lecona, Isabel Baeza, Fidel Martinez-Gutierrez, Erik Márquez-Gutiérrez, Gabriel Martínez-Castañon, Roberto Sánchez-Sánchez

**Affiliations:** 1 Laboratorio de Biotecnología, Instituto Nacional de Rehabilitación Luis Guillermo Ibarra Ibarra, Mexico City 14389, Mexico; minaorsa@hotmail.com (C.O.-S.); marioprz0586@gmail.com (M.P.-D.); yaazielmr@gmail.com (Y.M.-R.); ziorix@hotmail.com (N.Z.-J.); 2 Laboratorio de Biomembranas, Escuela Nacional de Ciencias Biológicas, Instituto Politécnico Nacional, Mexico City 07738, Mexico; isabelbaeza@yahoo.com; 3 Laboratorio de Tejido Conjuntivo, Instituto Nacional de Rehabilitación Luis Guillermo Ibarra Ibarra, Mexico City 14389, Mexico; bemmarcd@yahoo.com.mx; 4 Unidad de Ingeniería de Tejidos Terapia Celular y Medicina Regenerativa, Instituto Nacional de Rehabilitación Luis Guillermo Ibarra Ibarra, Mexico City 14389, Mexico; phaedrasilva@yahoo.com (P.S.-B.); val_mart76@yahoo.com.mx (V.M.-L.); 5 Laboratorio de Líquido Sinovial, Instituto Nacional de Rehabilitación Luis Guillermo Ibarra Ibarra, Mexico City 14389, Mexico; yesszamudio@gmail.com; 6 Departamento de Alimentos y Biotecnología, Facultad de Química, Universidad Nacional Autónoma de México, Mexico City 04510, Mexico; em.carmenhdz@gmail.com; 7 Laboratorio de Infectología, Instituto Nacional de Rehabilitación Luis Guillermo Ibarra Ibarra, Mexico City 14389, Mexico; esaulopezjacome@gmail.com; 8 Laboratorio de Microscopía Electrónica, Instituto Nacional de Rehabilitación Luis Guillermo Ibarra Ibarra, Mexico City 14389, Mexico; betocarlosmar@gmail.com (A.C.-M.); nena.1003@hotmail.com (N.R.-M.); 9 Cirugía Plástica, Instituto Nacional de Rehabilitación Luis Guillermo Ibarra Ibarra, Mexico City 14389, Mexico; luis687@hotmail.com; 10 Banco de Tejidos Radioesterilizados, Instituto Nacional de Investigaciones Nucleares, Ocoyoacac 52045, Mexico; esther.martinez@inin.gob.mx (M.E.M.-P.); lourdes.reyes@inin.gob.mx (M.d.L.R.-F.); 11 Bioterio, Instituto Nacional de Rehabilitación Luis Guillermo Ibarra Ibarra, Mexico City 14389, Mexico; lecona90@hotmail.com; 12 Laboratorio de Antimicrobianos, Biopelículas y Microbiota, Facultad de Ciencias Químicas, Universidad Autónoma de San Luis Potosí, San Luis Potosí 78210, Mexico; fidel@uaslp.mx; 13 Centro de Investigación en Ciencias de la Salud y Biomedicina, Universidad Autonoma de San Luis Potosi, San Luis Potosi 78210, Mexico; 14 Facultad de Estomatología, Universidad Autónoma de San Luis Potosí, San Luis Potosí 78210, Mexico; 15 Escuela de Ingeniería y Ciencias, Tecnológico de Monterrey, Mexico City 64849, Mexico

**Keywords:** radiosterilized pig skin, silver nanoparticles, fibroblasts, keratinocytes, burns, pre-clinical and clinical pilot study

## Abstract

Radiosterilized pig skin (RPS) has been used as a dressing for burns since the 1980s. Its similarity to human skin in terms of the extracellular matrix (ECM) allows the attachment of mesenchymal stem cells, making it ideal as a scaffold to create cellularized constructs. The use of silver nanoparticles (AgNPs) has been proven to be an appropriate alternative to the use of antibiotics and a potential solution against multidrug-resistant bacteria. RPS can be impregnated with AgNPs to develop nanomaterials capable of preventing wound infections. The main goal of this study was to assess the use of RPS as a scaffold for autologous fibroblasts (Fb), keratinocytes (Kc), and mesenchymal stem cells (MSC) in the treatment of second-degree burns (SDB). Additionally, independent RPS samples were impregnated with AgNPs to enhance their properties and further develop an antibacterial dressing that was initially tested using a burn mouse model. This protocol was approved by the Research and Ethics Committee of the INRLGII (INR 20/19 AC). Transmission electron microscopy (TEM) and dynamic light scattering (DLS) analysis of the synthesized AgNPs showed an average size of 10 nm and rounded morphology. Minimum inhibitory concentrations (MIC) and Kirby–Bauer assays indicated that AgNPs (in solution at a concentration of 125 ppm) exhibit antimicrobial activity against the planktonic form of S. aureus isolated from burned patients; moreover, a log reduction of 1.74 ± 0.24 was achieved against biofilm formation. The nanomaterial developed with RPS impregnated with AgNPs solution at 125 ppm (RPS-AgNPs125) facilitated wound healing in a burn mouse model and enhanced extracellular matrix (ECM) deposition, as analyzed by Masson’s staining in histological samples. No silver was detected by energy-dispersive X-ray spectroscopy (EDS) in the skin, and neither by Inductively Coupled Plasma Mass Spectrometry (ICP-MS) in different organs of the mouse burn model. Calcein/ethidium homodimer (EthD-1), 3-(4,5-dimethylthiazol-2-yl)-2,5-diphenyl tetrazolium bromide (MTT), and scanning electron microscopy (SEM) analysis demonstrated that Fb, Kc, and MSC could attach to RPS with over 95% cell viability. Kc were capable of releasing FGF at 0.5 pg above control levels, as analyzed by ELISA assays. An autologous RPS-Fb-Kc construct was implanted in a patient with SDB and compared to an autologous skin graft. The patient recovery was assessed seven days post-implantation, and the patient was followed up at one, two, and three months after the implantation, exhibiting favorable recovery compared to the gold standard, as measured by the cutometer. In conclusion, RPS effectively can be used as a scaffold for the culture of Fb, Kc, and MSC, facilitating the development of a cellularized construct that enhances wound healing in burn patients.

## 1. Introduction

Burns represent a significant global health burden, with an estimated 180,000 deaths attributed to burns worldwide according to the World Health Organization’s 2018 report. Most of these deaths occurred in low- and middle-income countries, where limited access to advanced medical technology might hinder the effective treatment of burn patients. The gold standard for treating deep second- and third-degree burns is a skin autograft; however, there is a scarcity of healthy skin, since normally there is a limited number of sites in the burn patient from which healthy skin can be harvested, and it is necessary to wait for the donor site to heal before tissue can be harvested again. Since Green’s publication on keratinocytes (Kc) in 1975, numerous protocols have been developed for in vitro skin tissue development [1]. In the United States and Europe, several companies commercialize these products, which typically combine fibroblast (Fb) for dermal development and Kc for epidermal development, both types of cells contribute to establishing the skin and also to release Fibroblast Growth Factor (FGF) and Epidermal Growth Factor (EGF) which are crucial growth factors for wound healing [2,3].

Tissue engineering products can offer an effective solution for treating burn patients when enough autograft donor sites are unavailable. Thus, developing accessible products in low- and middle-income countries is imperative. Since the 1980s, radiosterilized pig skin (RPS) has been utilized as a dressing to prevent water loss and infections in burn patients [4,5]; although it does not contribute to tissue repair, the extracellular matrix of RPS is similar to that of human skin, and its collagen has been demonstrated to be an excellent scaffold for cell viability, migration, proliferation, and growth factors release [6]. Moreover, RPS can serve as a scaffold for mesenchymal stem cells (MSC) culture, which has been shown to aid in wound healing by promoting extracellular matrix deposition in a burn mouse model [7]. Furthermore, RPS exhibits a significant advantage over human skin in terms of availability. Human tissues, including skin, face challenges due to the scarcity of organ donations. In contrast, RPS is more readily available and has been utilized since the 1970s. Interestingly, despite its long-standing use, RPS remains underexplored as a scaffold for cell culture or its functionalization for wound repair. This presents an exciting area of opportunity for its application in the field of tissue engineering. By harnessing the potential of RPS as a scaffold material, we can advance the development of innovative strategies in wound healing and tissue regeneration, providing promising alternatives to traditional approaches [8].

Infections are a significant concern when a burn occurs because the skin serves as the primary barrier against microorganisms. When the skin is destroyed, as it is in a burn injury, the wound is susceptible to infection by any microorganism, including multidrug-resistant bacteria. It is estimated that in the United States 35,000 deaths alone are caused by multidrug-resistant bacteria each year [9]. Silver has been used for a long time to treat infections in burn patients [10], where silver nanoparticles (AgNPs) represent an alternative to the use of antibiotics. In recent years, different AgNPs have been developed that not only inhibit the growth of bacteria but also improve wound healing [11,12]. One of these products is already on the market and it contains nanocrystalline silver and has been shown to be a good antimicrobial agent and wound healing promoter in chronic wounds [13].

In light of this, RPS has been impregnated with AgNPs to create a nanomaterial that inhibits bacterial growth while also promoting MSC proliferation [14]. In the present study, the RPS-AgNPs nanomaterials were further tested studying their: (a) antibacterial capability against a nosocomial bacteria strain isolated from a patient with second-degree burns (SDB), and (b) potential use as an antibacterial cover in an in vivo burn mouse model, where the wound healing process was monitored and the potential bioaccumulation of silver was characterized. A cellular construct was developed utilizing RPS as a scaffold for the culture of human fibroblasts and keratinocytes (RPS-Fb-Kc) and cell viability and FGF release on the construct was studied in vivo. Further, an autologous RPS-Fb-Kc construct was developed and tested, covered by the RPS-AgNPs nanomaterial as a wound dressing, to treat a deep second-degree burn patient. The biophysical parameters of the skin in the patient treated were measured over time by a cutometer assessing the elasticity, moisture, melanin, and erythema.

## 2. Materials and Methods

### 2.1. Isolation and Culture of Fibroblast, Keratinocytes, and Mesenchymal Stem Cells

The protocol for Fb and Kc isolation and their use was reviewed and approved (with number INR 20/19) by the Research and Ethics Committee of the Instituto Nacional de Rehabilitación Luis Guillermo Ibarra Ibarra (INRL-GII; Mexico). Inclusion criteria for patients in the protocol were: being 18–65 years, having no autoimmune pathologies, having a Total Burn Surface Area (TBSA) larger than 30%, having deep second-degree or third-degree burns, and providing signed Informed Consent. Exclusion criteria included: skin pathologies, autoimmune diseases, a history of cancer or diabetes mellitus, previous treatments with biological substitutes, or the presence of active infection in the burn area. Patients’ participation consisted of allowing the taking of a skin biopsy and the possible latter implantation of an autologous RPS-Fb-Kc construct covered by an RPS-AgNPS nanomaterial to treat a carefully chosen burn site. Once the patient agreed to participate in the study and signed Informed Consent, the clinician took a 1 cm^2^ skin biopsy from a non-burn area, and cell isolation was performed as previously described [15]. Briefly, the skin biopsy was washed with PBS with 10% penicillin/streptomycin (Gibco, Billings, MT, USA), and incubated in dispase (Gibco; 3 mg/mL) for 60 min. After incubation, the epidermis and dermis layers were separated and independently processed. To isolate Kc, the epidermal layer was incubated for 1 h in Trypsin/EDTA 0.25% (Gibco), then, the cell suspension was centrifuged, and isolated Kc were obtained after enzymatic inactivation. Kc were cultured at a density of 10,000 cells/cm^2^ on a feeder layer consisting of human Fb treated with Mitomycin C (Sigma, St. Louis, MO, USA; 10 mg/mL). DMEM/F12 supplemented with 1% Fetal Bovine Serum (FBS, Gibco) was initially used as a culture medium. After 3 days of culture, the medium was changed to 1% Human Keratinocyte Growth Supplement (Gibco). To isolate Fb, the dermal layer was mechanically disaggregated with a scalpel and enzymatically disaggregated for 2 h in type II collagenase (Gibco; 3 mg/mL). The cell suspension was centrifuged, and after inactivation of collagenase with DMEM/F12 supplemented with 10% FBS, Fb were obtained, counted, and cultured at a density of 10,000 cells/cm^2^, using DMEM/F12 medium supplemented with 10% FBS and 1% antibiotic-antimycotic (Gibco). Fb and Kc were cultured at 37 °C in a 95% humidity atmosphere with 5% CO_2_.

The protocol for adipose tissue samples’ procurement and MSC isolation was reviewed and approved (with number INR 78/15) by the Research and Ethics Committee of the INR-LGII (Mexico). Adipose tissue was obtained from young patients undergoing cosmetic surgeries via lipoaspirate who had signed Informed Consent. The procedure previously described by Sánchez et al. [6] was used for MSC isolation. Briefly, 20 g of adipose tissue was washed 3 times with PBS with 10% penicillin/streptomycin (Gibco) and subjected to mechanical and enzymatic disintegration with 300 μL (100 mg/mL) of type I collagenase (Worthington Biochemical, Lakewood, NY, USA) in 10 mL of DMEM (Gibco) under constant stirring for 45 min at 37 °C. Digested tissue was passed through a 70 μm filter and centrifuged at 1500 rpm. Cells were collected, resuspended, and seeded at a density of 10,000 cells/cm^2^. Adherent cells were expanded to passage 2 and characterized by flow cytometry with a FACSCalibur cytometer (FACS; Becton Dickinson, San Jose, CA) with a 488 nm blue laser. Three stem markers (+) coupled to a fluorochrome were analyzed: hCD90-FITC, hCD73-AP and hCD105-PE (BD Pharmingen, San Jose, CA, USA); as well as three hematopoietic markers (−): hCD45-FITC, hCD34-PE, and hCD166-PE (BD Pharmingen, San Jose, CA)). Finally, the data were analyzed with the Cell Quest Pro software v5.2.1 (Becton Dickinson Immunocytometry Systems, San Jose, CA, USA).

### 2.2. Radiosterilized Pig Skin

The radiosterilized pig skin used in this study was provided by the Banco de Tejidos Radioesterilizados (BTR, Toluca, Mexico) in Mexico City, which is part of the Instituto Nacional de Investigaciones Nucleares (ININ, La Marquesa, Mexico). The BTR has held a sanitary license for tissue processing since 6 July 1999 and is certified by ISO 9001:2000. Since 2001, the BTR has processed and radiosterilized pig skin, and the resulting tissues have been successfully utilized as wound dressings for patients at several hospitals in Mexico. The RPS processing protocol involves selecting the animals at an authorized slaughterhouse, followed by washing, drying, cutting, packing, labeling, and finally the sterilization of the RPS using ININ’s industrial ^60^Co gamma irradiator JS-6500 AECL at 25 kGy. All processing and sterilization stages are performed following the corresponding Standard Operation Procedure. After being submitted by a sterility test as a final product, the RPS was used in the present experiments.

### 2.3. Development and Study of the RPS-AgNPs Antibacterial Cover

#### 2.3.1. Development of the Nanomaterial; RPS-AgNPs Antibacterial Cover

The AgNPs synthesis and the development of the RPS-AgNPs covers were performed as previously described [14]. Briefly, for AgNPS synthesis, 100 mL of AgNO_3_ solution (10 mM) was mixed with 0.1 g of gallic acid in 10 mL of deionized water, pH was immediately adjusted to 11, then AgNPs obtained were purified by dialysis and characterized by zeta potential, Dynamic Light Scattering (DLS), and Transmission Electron Microscopy (TEM). Silver nanoparticles were characterized in aqueous solution using Dynamic Light Scattering (DLS) analysis by using a Malvern Zetasizer Nano ZS (Instruments Worcestershire, UK) operating with a He–Ne laser at a wavelength of 633 nm, and a detection angle of 90 degrees; nanoparticles were analyzed for 60 s at 25 °C. For the antibacterial cover development, RPS samples (10 cm × 10 cm) were independently incubated in 50 mL of 1000, 500, 350, 250, and 125 ppm AgNPs suspensions using 40 kHz sonication for 10 min; then, further incubation was performed for 24 h at room temperature in an orbital shaker at 250 rpm. Antibacterial covers were dried in a type A2 biological safety cabinet for 2 h and characterized in its topography and silver content by electron-dispersive X-ray spectroscopy (EDS) and SEM. Antibacterial covers were named as RPS-AgNPs1000, RPS-AgNPs500, RPS-AgNPs350, RPS-AgNPs250, and RPS-AgNPs125, according to the AgNPs’ concentration of the solution used for their preparation. The impregnation of silver in the RPS-AgNPs was previously analyzed using an air-acetylene flame atomic absorption spectrometer (Pinaacle 500, Perkin Elmer, Waltham, MA, USA), the atomic percentage for RPS samples impregnated with 125 ppm AgNPs solution; that is, RPS-AgNPs125 samples, was 0.59 ± 0.28 at.% [14].

#### 2.3.2. In Vitro Antibiofilm Capability

A nosocomial bacteria strain capable of adhering and developing biofilms was selected to evaluate in vitro the antibiofilm capability of the antibacterial covers. This strain was isolated from a patient with second-grade degree burns, following the guidelines of the American Society of Microbiology. The clinical strain identification and antimicrobial profile were determined by the automatized Vitek^®^ equipment. The antibiofilm capability of the antibacterial covers, RPS-AgNPs, and of RPS without AgNPs as a negative control, was evaluated using a slightly modified colony biofilm model [16]. Samples of 1 cm in diameter of RPS with and without AgNPs were examined. Suspensions of AgNPs were prepared to the following concentrations: 1000, 500, 350, 250, and 125 ppm, which were used to impregnate the RPS [14]. Circle samples of 1 cm diameter of the different antibacterial covers, and of RPS without AgNPs as a negative control, were independently placed on trypticase soy agar plates and inoculated with 10 mL of a high bacterial concentration suspension. Standardization of the inoculum was achieved using the pure and exponential growth phase of *Staphylococcus aureus* (to the McFarland 0.5 scale; 0.08 to 0.1 absorbance at 600 nm) diluted 1:1000 to approximately obtain 1.5 × 10^5^ colony-forming units per mL (CFU/mL); actual concentration of microorganisms in the inoculum was corroborated by CFU counting on trypticase soy agar plates. After 24 h of incubation at 37 °C, mature biofilms were recuperated from the inoculated antibacterial cover samples, all samples were rinsed with 180 mL of saline solution, and independently placed in glass tubes with 9 mL of saline solution. Biofilm disaggregation was carried out with a combination of low-intensity sonication and vortexing according to Goeres et al. [17]. Homogeneous supernatants were used to determine the concentration of microorganisms by CFU counting on trypticase soy agar plates, according to the Miles–Misra method. CFU/mL numbers were recorded and log10-transformed. Log densities were converted into a measure of the log reduction to determine the antibiofilm efficacy. Log reduction was calculated as the mean log density for the RPS without AgNPs minus the mean log density for the corresponding RPS-AgNPs antibacterial cover [18]. Mean averages and standard deviations (SD) are reported.

#### 2.3.3. In Vivo Evaluation; Athymic Mouse Burn Model

##### Burn Wound Animal Model

According to the present results on the antibiofilm capability, and previously reported results on the in vitro biocompatibility [14] of the antibacterial covers developed with different AgNPs concentrations, the antibacterial cover developed by using 125 ppm AgNPs solution was chosen as the antibacterial cover with the most potential (RPS-AgNPs125), and its biocompatibility was further evaluated in vivo using a burn mouse animal model. In vivo biocompatibility evaluation protocol was approved by the Animal Care Committee and the Research Committee of the INR-LGII with register number INR 78/15. Next, 40 NU/NU nude male mice with an average age of 8 to 9 weeks were provided by the Production and Experimentation Unit of Laboratory Animals (UPEAL) of the Center for Research and Advanced Studies (CINVESTAV) of the National Polytechnic Institute in Mexico. Mice were delivered with a health certificate, ensuring they were free of pathogenic agents. Upon arrival to the bioterium of the INR-LGII, mice remained in a two-week adaptation and quarantine period in LifeSpan^®^ cages in a room exclusively for immunosuppressed animals, constantly monitored with health and behavior evaluations, as well as periodic changes of cage, food, and sterile water; always handled in a laminar flow hood. After the adaptation period, mice were divided into 8 different groups (*n* = 5), corresponding to each of the treatments and time periods to be evaluated. Treatments corresponded to Gauze, RPS, RPS-AgNPs125, and AgNPs in 1000 ppm suspension, and days for mice sacrifice and histological evaluation corresponded to 7 and 14 days after treatment per treatment group.

For wound burn induction (Figure S1), each mouse was anesthetized (6% isoflurane induction dose), weighed under a state of unconsciousness, and transferred to the surgery table in an operating room under sterile conditions. Burn injury was manually performed on the back of the mouse using an apparatus with a weight of 366 g and a circular flat copper tip of 2 cm^2^ diameter. The tip was heated to 225.62 ± 7.791 °C using an alcohol burner, and then it was placed in contact with the back of the mouse for 5 s; tip temperature reproducibility was determined by taking 25 duplicate temperature measurements. These conditions resulted in a deep second-degree burn injury. Immediately after burn induction, the injured area was photographed as day 0 of treatment, the wound burn was debrided, and corresponding treatment was applied, that is, gauze as no treatment control, RPS, RPS-AgNPs125, or AgNPs in 1000 ppm suspension. Then, Hypafix^®^ was bandaged around the mouse torso to cover and secure the treatment and prevent its detachment. Finally, mice were placed on a thermal mattress and a butorphanol unit was administrated as an analgesic. Upon mice regaining consciousness and recovering mobility, they were returned to their confinement boxes in the area for immunosuppressed animals (Figure S1).

##### Macroscopic Evaluation of Wound Closure

To macroscopically evaluate burn closure, the burn area was measured on days 0, 3, 7, 10, and 14. The pictures were taken using a professional Nikon digital camera mounted on a tripod 60 cm above the mouse. To immobilize the mouse and acquired photographs, a sustained dose of isoflurane at 6% was applied. Upon losing consciousness, the mouse was weighed and the Hypafix bandage removed. Once photographs were taken, the mouse was re-bandaged with Hypafix and returned to its confinement cage. Photographs were high-resolution and analyzed using Image-J software v 1.53e, in which a metric reference was incorporated to establish the pixel to cm ratio. Once the pixel:cm ratio was stablished, the burn perimeter was measured and the burn area was calculated. A *p*-value less than 0.05 was considered statistically significant.

##### Microscopic Evaluation of Wound Repair

A total of 5 mice per group of treatment were sacrificed at days 7 and 14 after burn induction to evaluate skin repair (and possible AgNPs bioaccumulation in the main blood-supplied organs). Mice were sacrificed in a CO_2_ chamber. Then, skin biopsies from the wound area were taken by surgically cutting with scissors and a scalpel a square sample with a 3 mm margin around the burn wound. Skin biopsies were flattened in a natural way (no over or under stretching) on a paraffin surface, fixing them with pins to prevent them from contraction, fixed with 4% paraformaldehyde (PFA) for 24 h and divided in two parts by the middle of the burn wound for microscopic evaluation of wound repair (histological staining) and analysis of potential accumulation of silver on the wound (SEM and EDS analysis). Skin samples for SEM and EDS evaluation were washed three times with PBS, dehydrated through a series of graded increasing ethanol solutions, dried overnight, and sputtered with Au thin films before the backscattered electron’s SEM observation and EDS elemental analysis at 10 kV (JEOL 7600). Skin samples for histological analysis were processed in a Thermo Scientific (Waltham, MA, USA) Histoquinete Microm STP-120 for clearance through dehydration with increasing concentration graded ethanol solutions, xylol and paraffin, and finally, individually paraffin-embedded. Then, 5 μm thick sections were obtained from embedded samples (Leica microtome, Wetzlar, Germany), mounted on a glass slide coated with TESPA, placed in a heating furnace, and subjected to rehydration through a series of descending concentration (down to 50%) ethanol solutions. Hematoxylin–eosin (H&E) staining of skin sections was used for general assessing of microscopic skin structure and staining cell nuclei in blue and cytoplasm and extracellular matrix in pink. Masson’s trichrome staining was used to evaluate skin collagen deposition, staining collagen in blue, cell nuclei in black, and cell cytoplasm in red. Briefly, for H&E staining, rehydrated skin section samples were immersed in a Harris hematoxylin solution, rinsed with tap water, transferred to 1% acid alcohol solution, rinsed with tap water again, immersed in lithium carbonate solution, transferred to 80% ethanol solution, placed in eosin solution, gradually dehydrated in ethanol increasing concentration solutions and xylol, and finally, mounted with resin and analyzed with an Axio Imager Z2 (Zeiss, Oberkochen, Germany) microscope. For Masson’s trichrome staining, independent rehydrated skin section samples were rinsed in Bouin’s solution, washed with water, immersed in Weigert’s hematoxylin, rinsed in a Biebrich’s scarlet solution, transferred to Masson’s acids, washed in water again, immersed in aniline blue solution in acetic acid, and finally, dehydrated in increasing concentration ethanol solutions and xylol and mounted with Entellan for further examination with an Axio Imager Z2 (Zeiss) microscope.

##### Determination of Potential Silver Bioaccumulation in Burned Skin and Organs in the Animal Model

To determine the possible biodistribution of silver in the organism of the mouse, heart, spleen, pancreas, and liver were carefully dissected after sacrificing the mice and the taking of skin biopsies. Organs were dissected by carefully removing the connective/adipose tissue around them, and then, they were individually stored in 2 mL Eppendorf tubes at −80 °C until experimental processing. Potential silver presence in dissected organs at 7 days after burn wound induction was analyzed by mass spectrometry. As a first step, the exact weight of each organ of interest was determined. Subsequently, organs were individually digested in a vessel containing 5 mL of concentrated HNO_3_ solution at 60 °C. Once the organs were completely digested, the temperature was raised to 100 °C to evaporate the remaining nitric acid. The walls of the vessel were carefully washed with HCl to remove the possible remaining silver ions from the organs’ digestion, and finally, the obtained solution was diluted and calibrated to a known volume to be analyzed by Inductively Coupled Plasma Mass Spectrometry (ICP-MS; Thermo X series II). The results are reported in silver ppm.

### 2.4. Cellular Construct In Vitro Studies

#### 2.4.1. Cellular Constructs

Independent cellular constructs were generated to study the biocompatibility and performance of RPS as a potential scaffold to generate a combined cellular construct (RPS-Fb-Kc) for burn wound treatment. Three different independent cellular constructs were generated by using Fb, Kc, and MSC isolated from patients’ skin biopsies and lipoaspirates, respectively. Isolated Fb, Kc, and MSC were trypsinized and independently seeded on RPS samples at a density of 30,000 cells/cm^2^. Cell seeding on RPS was performed using DMEM/F12 medium with 1% of penicillin/streptomycin and 10% of FBS, with cells suspended in 10 µL (30,000 cells) drops of medium that were seeded on the RPS samples. Constructs were incubated for 1 h at 37 °C, 95% humidity, and 5% CO_2_. After incubation, fresh medium was added to the culture petri dishes containing the constructs until it completely covered them, and constructs were further incubated for 24 h.

#### 2.4.2. Cell Viability and Adhesion

Cell viability on the constructs was analyzed at 24 h of culture using calcein and ethidium homodimer assays (Live/Death Viability/Cytotoxicity for mammalian cells kit; Invitrogen (Waltham, MA, USA), following the specifications of the manufacturer. Briefly, the constructs were washed with 1X PBS (Gibco) and incubated in 1 μM calcein AM and 2 μM ethidium homodimer (EthD-1) diluted in Hank’s medium for 1 h. After incubation, the constructs were examined under an epifluorescence microscope (Zeiss) and photographs were acquired using the AxioVision Software v 2.6. Controls (CTL) corresponded to cells seeded and cultured on standard tissue culture plates.

Independent construct samples (after 24 h of cell culture on RPS) were fixed in glutaraldehyde 5% and dehydrated through a series of graded increasing ethanol solutions, dried overnight, and sputtered with Au thin films for SEM observation of cells adhered to the RPS scaffold.

#### 2.4.3. Potential Cytotoxicity of RPS

Potential cytotoxicity of RPS against Fb and Kc was also evaluated in the Fb-RPS and Kc-RPS constructs at 24 h of culture by using the colorimetric MTT (3-(4,5-dimeth-yl-thiazol-2-yl)-2,5-diphenyl tetrazolium bromide) assay. Briefly, after 24 h of culture constructs were rinsed with PBS and incubated with MTT:DMEM solution (1:10) for 3 h. Then, cells metabolized formazan crystals were solubilized in 2-propanol:dimethyl sulfoxide (1:1) and dissolution’s absorbance was measured at 570 nm (Synergy HTX spectrophotometer). Experiments were independently performed in triplicate. Controls (CTL) corresponded to cells seeded and cultured on standard tissue culture plates.

#### 2.4.4. Cellular FGF Expression on RPS

To determinate whether RPS induces the release of FGF from Kc, ELISA assays were performed using a specific kit for FGF evaluation (Peprotech; catalogo). RPS samples were seeded with human-isolated Kc at 30,000 cells/cm^2^ (using DMEM/F12) and cultured for 24 h. Then, 200 μL of supernatants from culture experiment were placed in a microplate previously coated for 24 h with the capture antibody. The cell supernatants were incubated for 2 h, and each well was washed with phosphate buffer (PBS/Tween). Post-incubation, the detection antibody was added, and the plates were incubated for 2 h at room temperature. Finally, avidin-HRP and ABTS substrate solutions were added, and the plate was further incubated for 30 min. The absorbance was measured at 405 nm, and results were compared to a standard curve of the kit.

### 2.5. Case of Study: Autologous Cellular Construct Dressed with Antibacterial Cover

To evaluate the potential of the cellular construct RPS-Fb-Kc in combination with the RPS-AgNPs125 antibacterial cover as a complete treatment for second-degree burn patients, an autologous cellular RPS-Fb-Kc construct was generated, implanted in a burn patient, and dressed with the RPS-AgNPs125 antibacterial cover.

#### 2.5.1. Autologous Cellular Construct Generation

Fb and Kc were isolated from a skin biopsy of the patient to be treated and in vitro expanded according to the protocol with Research and Ethic Committee approval number INR 20/19 mentioned in Section 2.1. Seeding of autologous Fb and Kc on RPS was performed as mentioned in Section 2.4.1 generating a square 10 cm × 10 cm autologous RPS-Fb-Kc cellular construct. The autologous RPS–Fb–Kc construct was incubated for 1 h at 37 °C, 95% humidity, and 5% CO_2_, then, fresh culture medium was added to the culture petri dish containing the RPS–Fb–Kc construct in a sufficient amount to completely cover the construct; and then it was further incubated for 24 h before implantation in the burn patient.

#### 2.5.2. Cell Viability on Autologous Construct

Cell viability was analyzed on the day of the autologous cellular construct implantation on the patient using one square centimeter of the developed autologous RPS–Fb–Kc construct. Calcein and ethidium homodimer assay was performed using the Live/Death Viability/Cytotoxicity for mammalian cells kit (Invitrogen), following the specifications of the manufacturer. Briefly, the autologous construct sample was washed with 1× PBS (Gibco), incubated in 1 μM calcein AM and 2 μM ethidium homodimer (EthD-1), and diluted in Hank’s medium for 1 h. After incubation, the autologous construct sample was examined under an epifluorescence microscope (Zeiss) and photographs were acquired using the AxioVision Software v 2.6.

#### 2.5.3. Microbiological Test for the Construct to Be Implanted in the Patient

The Microbiological testing of the autologous cellular construct to be implanted was performed at the Laboratorio de Infectología at the INRLGII by taking samples of the culture medium at different stages during the generation, and right before implantation, of the autologous cellular construct. That is, medium samples were taken when the skin biopsy to isolate Fb and Kc was taken from the patient, during autologous cells culture, and from the incubation medium of the autologous RPS-Fb-Kc construct right before implantation. Microbiological testing was also performed on the RPS-AgNPs125 antibacterial cover by taking samples of the medium used to generate the cover. For microbiological analysis, samples were independently inoculated onto 5% blood sheep, MacConkey, dextrose Sabouraud, and phenylethyl alcohol with 5% sheep blood agar (PEA). PEA was incubated in anaerobic atmosphere within Anaerobe GasPak System (Becton-Dickinson, Franklin Lakes, NJ, USA) at 37 °C, dextrose Sabouraud was incubated at room temperature, while 5% blood sheep agar and MacConkey were incubated at 37 °C under aerobic conditions. An aliquot of the samples was also inoculated into a pediatric bottle and incubated in a Bactec system (Becton–Dickinson, EEUU) for 8 days. Plates were observed and reviewed to verify the absence of microbial growth.

#### 2.5.4. Implantation of the Autologous Cellular RPS-Fb-Kc Construct and Cover with the RPS-AgNPs125 Nanomaterial

The patient was a 50-year-old female that had burns by fire and she accepted to sign the consent letter and participate in the study, she had 31 percent of the burn total body surface area, with second- and third-degree burns in face, neck, thorax, abdomen, back, and both upper and lower limbs. Most of the burns were treated with tangential excision and autografts and two zones or approximately three-square centimeters were treated with the autologous construct. The autologous RPS–Fb–Kc construct developed was implanted on the patient posterior to the autologous skin autograft (gold standard treatment); the construct was implanted within a deep second-degree burn. Simultaneously, the RPS-AgNPs125 was applied over the construct to prevent infections. After seven days, the burn wound was uncovered and documented with photographs.

#### 2.5.5. Clinical Follow-Up of the Patient

The patient was monitored at 1, 2, and 3 months post-surgery. On the day of the follow up, the patient rested for at least 20 min in an isolated room before biomechanical skin properties evaluation. Measurements were conducted in a controlled-temperature (22 ± 1 °C) room with a relative humidity of 30 ± 5%, with the patient seated with her arms and palms extended in supine position. Biomechanical skin properties were assessed using a dual MPA 580 cutometer (CK electronic GmbH, Köln, Germany) with a 2 mm diameter probe. Measurements were carried out in a time-strain mode (Modus 1) with 10 cycles of 450 mbar of negative pressure for 2 s (on-time), followed by 2 s relaxation time (off-time). Skin mechanical parameters analyzed were R0, R5, and R6. R0 represents the extensibility and correlates to skin pliability/firmness; lower values represent higher firmness. R5 represents the net elasticity; higher values represent a more elastic skin. R6 represents the ratio of viscoelasticity to elasticity; lower values imply greater elasticity. R-values were analyzed using the cutometer software v. 2.0.0.1. The average value from 3 independent measurements was used for the analysis. Skin moisture was measured using a Corneometer CM825 (CK electronic GmbH), which measures the electrical capacitance of water in the stratum corneum in arbitrary units. The probe and skin were wiped with a dry cloth and five measurements were taken in succession. Pigmentation (melanin) and erythema index were measured with the Mexameter MX18 (CK electronic GmbH), which measures the light reflected by the skin. Values were analyzed using CK-MPA-Multi-Probe-AdapterFB v. 2.2.0.1 software. The average of five independent measurements was used for the analysis.

## 3. Results

### 3.1. AgNPs Characterization

**Figure 1:**
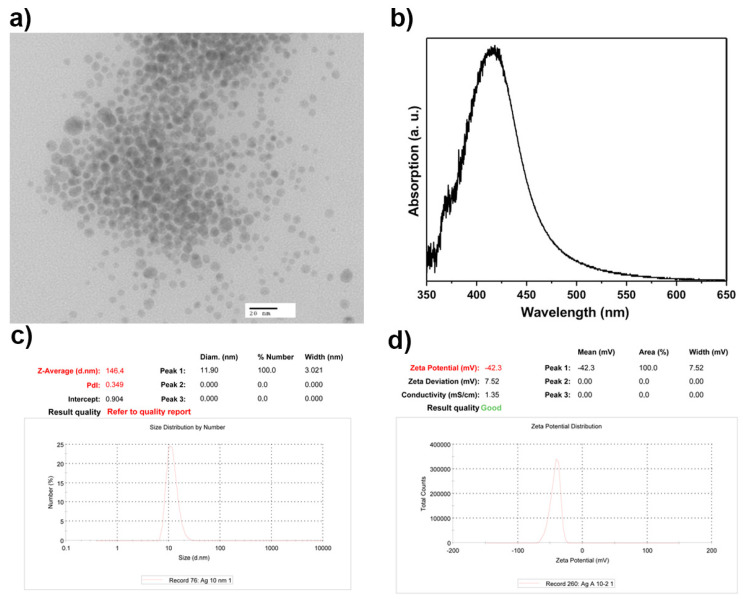
Characterization of silver nanoparticles. (**a**) Representative TEM microphotograph of the AgNPs. (**b**) Surface plasmon resonance observed at 420 nm. (**c**) Representative distribution curve of the hydrodynamic AgNPs diameter. (**d**) Zeta potential of the AgNPs.

Silver nanoparticles were characterized by zeta potential, DLS, and TEM. The results showed spherical nanoparticles with an average size of 10 nm, exhibiting a zeta potential of −42.3 mV, indicating long-term stability. Moreover, a surface plasmon resonance (SPR) at 420 nm was observed which is characteristic of silver nanoparticles (Figure 1).

### 3.2. Antibacterial Effect of the Nanomaterial; RPS-AgNPs Covers

To evaluate the antibacterial activity of the RPS impregnated with AgNPs at different concentrations, antibiofilm assays were performed using a nosocomial bacteria strain isolated from a SDB patient. Antibiofilm activity was AgNPs concentration dependent and biofilm log reduction increased as the concentration of the AgNPs increased; Table 1. The antibacterial cover developed by using the lowest concentration of AgNPs studied (125 ppm solution) showed significant antibiofilm activity against a representative clinical isolate of methicillin-resistant *S. aureus*, reducing biofilm formation in a log reduction of 1.74 in comparison to RPS with no AgNPs (Table 1). According to this result and previously obtained results [14], biocompatibility of AgNPs-impregnated RPS covers decreases as AgNPs concentration increases, the antibacterial cover developed by using the lowest concentration of AgNPs studied, RPS-AgNPs125, was chosen as the antibacterial cover for further in vitro and in vivo evaluation.

### 3.3. Characterization and Cell Viability Analysis of MSC on RPS-AgNPs125

Since MSC are a population of cells with the potential to improve wound healing through their differentiation to vascular tissue, release of VEGF, and immunoregulation properties, the capability of MSC to adhere to and be viable on RPS and RPS-AgNPs125 was analyzed. The MSC were isolated from adipose tissue, specifically from lipoaspirates containing the stromal vascular section, and characterized by flow cytometry on their expression of mesenchymal (+) and hematopoietic (−) stem markers. The isolated cells showed the characteristic markers of adipose-derived mesenchymal stem cells being positive for CD90 (97.98%), CD73 (96.62%), and CD105 (64.26%). On the other hand, they were negative for characteristic hematopoietic markers, CD45 (0.42%), CD34 (1.81%), and CD166 (1.13%) (Figure S2). The biocompatibility of a scaffold is an essential factor for tissue engineering or regenerative medicine applications. To assess and corroborate the biocompatibility of the RPS and that of the antibacterial cover RPS-125AgNPs, prior to the in vivo assay with athymic mice, MSC were seeded on RPS and RPS-AgNPs125 and its viability after 48 h of culture was evaluated. Viability tests performed with calcein and EthD-1 showed that both materials (RPS and RPS-AgNPs125) allow adhesion and cell viability of MSC. However, as expected, RPS-AgNPs125 significantly reduced cell adhesion in comparison to RPS with no AgNPs (Figure S2).

### 3.4. RPS Is a Permissive Scaffold for Fibroblasts, Keratinocytes, and MSC Growth

**Figure 2:**
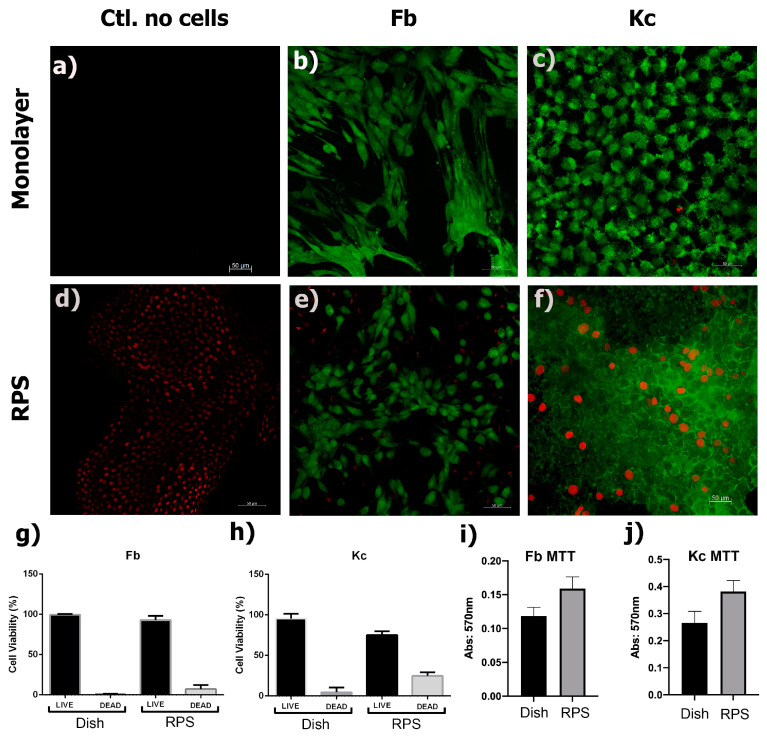
Viability assay of fibroblasts and keratinocytes on RPS. The photographs show cell viability analysis by calcein (live-green) and EthD-1 (dead-red), in the top panels (**b**,**c**) cells seeded in cell culture dishes as control are shown, and at the bottom panels (**e**,**f**), the cells seeded on RPS are shown. (**a**,**d**) show the photographs corresponding to RPS without cells. Graphs in g and h illustrate the percentage of live and dead (**g**) fibroblast and (**h**) keratinocytes, respectively, according to live/dead immunofluorescence assay in comparison with cells when cultured in monolayer (standard tissue culture plates). Graphs in (**i**,**j**) show the MTT assay results for (**h**) fibroblast and (**j**) for keratinocytes. In MTT assays, no significant differences were observed with the control in any case, using the Student’s *t*-test. Scale bar: 50 μm.

Kc and Fb are the main cell type of the skin and they are important mediators of the wound healing process [19,20]. In order to evaluate whether RPS could also serve as a scaffold for fibroblast and keratinocytes culture, these two cell types were independently seeded on RPS samples. In addition, Fb and Kc were cultured on standard tissue culture plates as a control. The results of the calcein/EthD-1 assay showed that fibroblasts and keratinocytes had more than 95% viability when cultured on the RPS (Figure 2e–h). An MTT assay was performed to analyze cellular metabolic activity upon culture on RPS. Fb and Kc demonstrated similar metabolic activity compared to the control; Figure 2i,j.

**Figure 3:**
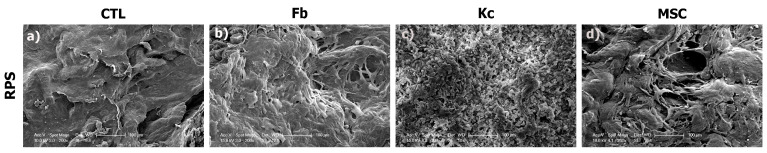
RPS allow the culture of fibroblast, keratinocytes, and MSC. The image shows micrographs taken by SEM, from left to right; (**a**) RPS with no cells (CTL), and (**b**) fibroblast, (**c**) keratinocytes, and (**d**) MSC cultured on RPS. Scale bars correspond to 100 µm.

Representative SEM micrographs of Fb, Kc, and MSC acquired after 24 h of culture on RPS are shown in Figure 3. SEM analysis shows the surface structure of RPS (Figure 3a), and the presence of well-adhered Fb, Kc, and MSC (Figure 3b–d) on the RPS surface. These results support the observations in the viability assays, corroborating that RPS is a good scaffold for the growth of skin and MSC cells to produce appropriate cellular constructs. As it is shown in the calcein/EthD-1 results (Figure 2), the morphology of the Fb on RPS is the expected spiculated morphology of adhered fibroblasts while Kc on RPS also show their characteristic adhered cells polygonal morphology. These morphologies are also corroborated by SEM images (Figure 3). This data shows that cells remain viable and maintain their characteristic morphologies of well-adhered cells when cultured on RPS.

**Figure 4:**
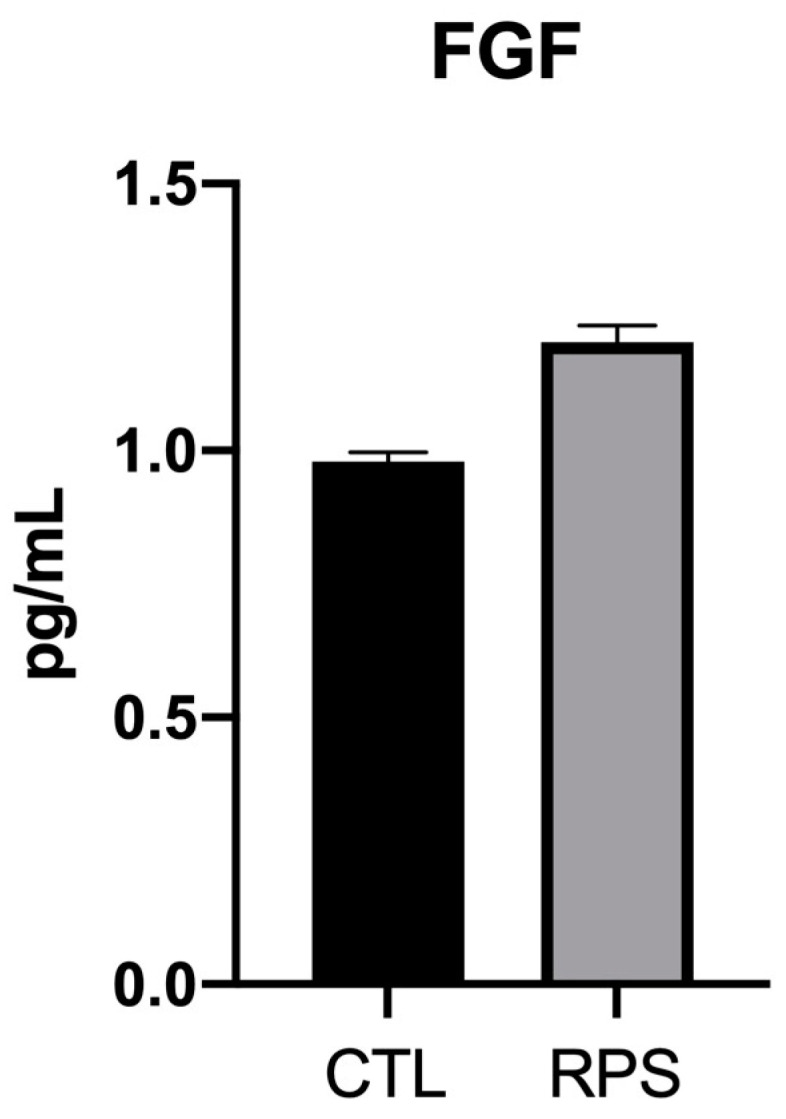
Determination of FGF from keratinocytes. The graph shows the concentration of FGF measured by ELISA under different experimental conditions. Kc cultured on RPS and standard tissue culture plates (CTL). ANOVA test. The bars correspond to standard error.

Additionally, Kc were seeded on RPS and culture for 24 h without FBS to analyze the fibroblast growth factor (FGF) release; Kc seeded on a tissue culture plate were used as a control (CTL). FGF is an essential factor that not only induces Kc proliferation but also stimulates the ECM synthesis in the skin, thus enhancing wound healing [21]. Our results showed that FGF expression from Kc cultured on RPS do not show significant differences in comparison to the cells seeded over cell culture dishes (Figure 4).

### 3.5. Radiosterilized Porcine Skin Impregnated with Silver Nanoparticles Promotes Wound Closure in a Burn Animal Model

**Figure 5:**
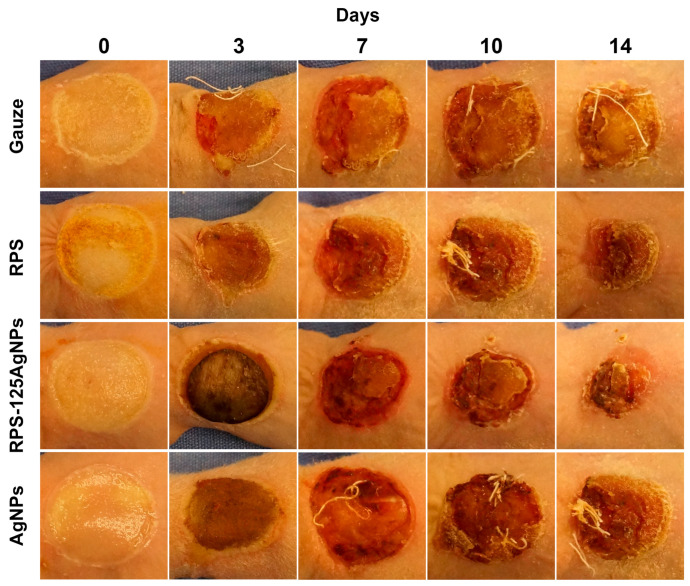
Macroscopic aspect of burn injury shows a better reduction with RPS-AgNPs125 treatment. After 14 days of surgery, treatments with RPS and RPS-AgNPs125 show a greater reduction in the area affected by the burn. The pictures were taken at 0, 3, 7, 10, and 14 days after burn induction to visualize the progression of healing. The photographs (*n* = 5) have the same resolution (300 dpi) and capture distance (30 cm). The Photoshop software (CS6) was used for standardization for equal cuts of 3.5 × 3.5 cm for each photograph.

To analyze the effect of RPS-AgNPs125 as wound dressing, a burn animal model was used to study the dressings effects in the wound healing process. Progression of the burn closure was monitored at 0, 3, 7, 10, and 14 days (Figure 5). During this period, no mouse deaths or exclusions were recorded from any experimental group. Experimental treatment groups were as follows: gauze, RPS, RPS-AgNPSs125, and AgNPs in suspension at a concentration of 1000 ppm. The Hypafix bandages placed over the treatments were removed and replaced on each scheduled day for evaluation of burn closure. Upon bandage removal, pictures were taking to evaluate the progress in burn closure. No malformations or accumulations of any kind of secretion, due to exudate or bacterial infection, were observed. Incorporation of nanomaterials, such as silver nanoparticles into the structuring of dermal substrates plays an important role and should not negatively interfere with the tissue repair process [22]. In some cases, the RPS or RPS-AgNPs125 covers studied remained attached to the burn wound until the seventh day of the treatment, where they finally detached without leaving any type of injury. Photographs revealed that the treatments with RPS and RPS-AgNPs125 resulted in an improved wound closure; at all monitoring days a smaller wound area was observed in the burn area and a better superficial skin appearance for RPS and RPS-AgNPs125 in comparison to the gauze or AgNPs in suspension treatments (Figure 5).

**Figure 6:**
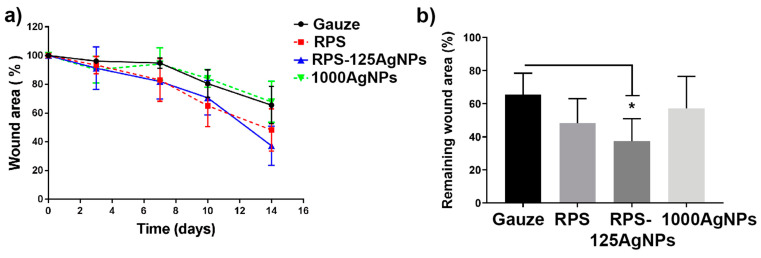
Radiosterilized pig skin impregnated with silver nanoparticles (RPS-AgNPs125) improves the wound closure process. The healing process was quantitatively evaluated from pictures of the burn wound taken at 0, 3, 7, 10, and 14 days post-treatment. The burn area (mean ± SD) was calculated with ImageJ software v 1.53e (*n* = 5 per treatment), and the data were transformed into a percentage. (**a**) the graph shows the percentage of healing area for all the experimental treatments over fourteen days. (**b**) the graph shows the remaining wound area on day 14 post-treatment; a significant effect was observed (* *p* < 0.05) for RPS and RPS-AgNPs125 in comparison with the other treatments evaluated. One-way non-parametric ANOVA (Kruskal-Wallis) was used to determine statistical significance.

The analysis of the burn area reduction was performed on days 0, 3, 7, 10, and 14 after burn induction, revealing a uniform trend in wound reduction for each one of the treatments. Similar behavior was observed in all treatments initially (day 3), while from day 7 and onward, RPS and RPS-AgNPs125 started showing a better trend for wound healing. In contrast, the treatment with AgNPs in suspension followed the same trend as the control (gauze) (Figure 6a). By day 14, marked differences were observed, with RPS and RPS-AgNPs125 treatments demonstrating a superior wound closure. The RPS-AgNPs125 treatment presented significant differences (*p* < 0.05) compared to the control (gauze), leaving a smaller remaining wound area in the burn zone (Figure 6b).

At seven and fourteen days post-treatment, reepithelization was observed under the crust of the wound area across all experimental conditions. The crust is marked with an asterisk in supplementary Figure S3. In all cases the epidermis was wide, due to the keratinocytes in high proliferation to repair the damage and no notable differences between the experimental groups were found using hematoxylin and eosin stains (Figure S3).

The Masson stain allows one to differentiate the keratin, stained in red, from the collagen, stained in blue. The histological analysis indicates that on the fourteenth day post-treatment, the presence of collagen in the new dermis under the re-epithelization process is only detected for the treatment with RPS and RPS-AgNPs125 (Figure S4). At seven days post-treatment, a subtle blue stain is observed under the newly formed epidermis, for both treatments, RPS and RPS-AgNPs125.

### 3.6. Analysis of AgNPs Deposition on Burned Skin and Possible Ag Bioacumulation in Organs from Mice Treated Independently with the RPS-AgNPs125 Antibacterial Cover or AgNPs Solution at a Concentration of 1000 ppm

One of the main concerns regarding the use of AgNPs is their potential accumulation in various organs and the consequent potential cytotoxic effects. Previous studies have shown that systemic, oral, and chronic exposure to AgNPs induces negative effects and bioaccumulation in the organs with the greatest blood supply [23,24]. In our study, the concentration of silver and AgNPs in internal organs (liver, spleen, kidney, and heart) and burn skin, was determined to analyze if traces of them remain upon athymic mice independently treated with RPS-AgNPs125 antibacterial cover or AgNps in suspension at a concentration of 1000 ppm. Exposure to silver nanoparticles in suspension was performed only once, while scaffolding of RPS-AgNPs125 depended on the time of adhesion to the wound area (approximately 7 days). The evaluation at day 7 of treatment showed the absence of accumulation of silver in the organs of interest measured by ICP-MS (Table 2).

**Figure 7:**
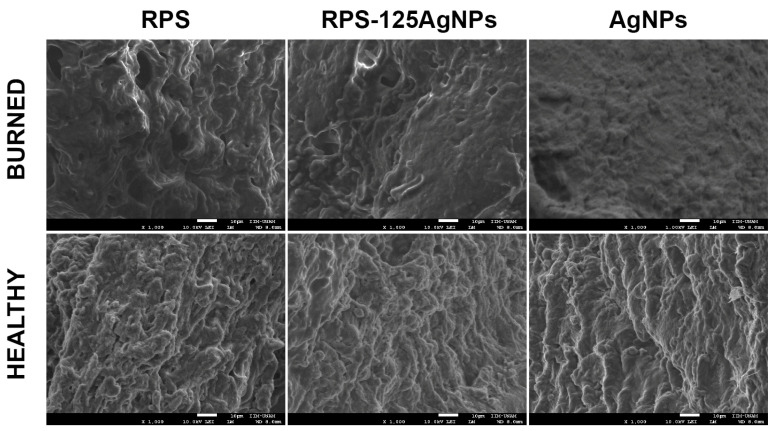
The treatment with RPS-AgNPs125 or AgNPs in suspension at 1000 ppm do not promote silver bioaccumulation in the burned area. The RPS, RPS-AgNPs125, and AgNPs samples were studied by EDS and SEM to characterize its elemental composition, morphology, and AgNPs surface distribution. The results show that there is no bioaccumulation of silver in the burn or healthy skin tissue. The analyses were acquired in a scanning electron microscope JEOL 7600 at 10 kV in secondary and backscattered electrons modes.

In the same way, the elemental analysis by SEM (backscattered electrons) of the mice burn skin showed a negative result for the presence of silver in the burn area (Figure 7). The nature of the skin burn (cauterization) possibly contributed to the null systemic distribution of AgNPs observed, since cauterization might limits the exposure of the system to the AgNPs.

### 3.7. Implantation of the Construct and Nm Application in a Clinical Pilot Study

**Figure 8:**
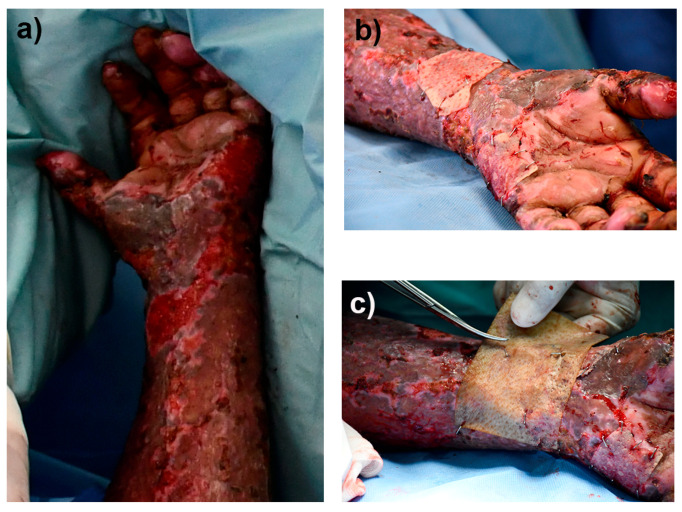
Implantation of the autologous cellular construct and dressing with the RPS-AgNPs125. Figure (**a**) shows the burns before the implantation of the autologous cellular construct, (**b**) the application of the autologous cellular construct, and (**c**) the use of the RPS-AgNps125 to cover the construct.

**Figure 9:**
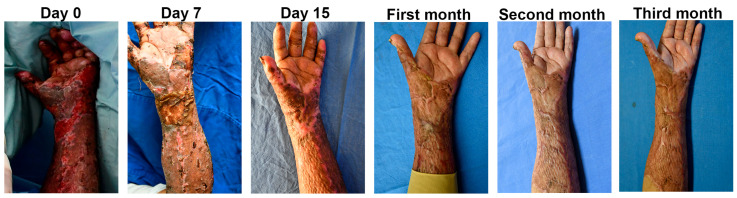
Following of the wound healing. The images show the following of the wound healing before the implant, at days 7 and 15 and at months one, two, and three.

One square centimeter of skin from the patient was used to obtain Fb and Kc, and the medium used to transport the skin sample was sent to be analyzed by the infectiology laboratory. At a clean room, the cells were isolated from the skin biopsy and expanded over two weeks, Fb were used at passage 2 and Kc at primary culture. An autologous cellular construct of 10 cm × 10 cm was developed by seeding on RPS, 30,000 Fb and 30,000 Kc per square centimeter of RPS (Figure S5a). In total, 1 cm^2^ of the autologous cellular construct was used to analyze the cell viability on the RPS using the calcein/EthD-1 assay that showed that most of the cells were viable at the time of the autologous cellular construct implantation (Figure S5b). Right before autologous cellular construct implantation, an RPS-AgNPs125 cover was developed as previously described [14] by impregnating a 10 cm × 10 cm RPS sample with AgNPs 125 ppm solution (Figure S5a). At the time of the implantation surgery the autologous cellular construct was washed with saline solution before its application in a second-degree burn. The construct was applied next to a zone previously treated with an autograft (Figure 8) and over the construct and the RPS-AgNPs125 was applied. Then, the patient was covered with gauze. The patient was uncovered 7 days later and photographs were taken (Figure 9). Following of the patient progress was performed at 15 days, and 1, 2, and 3 months after construct implantation.

On the seventh day, the burn area that was treated with the construct and covered with RPS-AgNPs125 was epithelized and the RPS-AgNPs125 cover fell off when the bandages were removed. On day fifteen, the skin looked pink, indicating that there was no melanin synthesis yet, but the wound was completely re-epithelized. At months one, two, and three the cutometer was used to analyze different physiological parameters of the skin. At this time, the wound was completely healed and melanin was observed at all the analyzed time points. The skin at the site treated with the construct was smoother in comparison with the autograft meshed (Figure 9).

**Figure 10:**
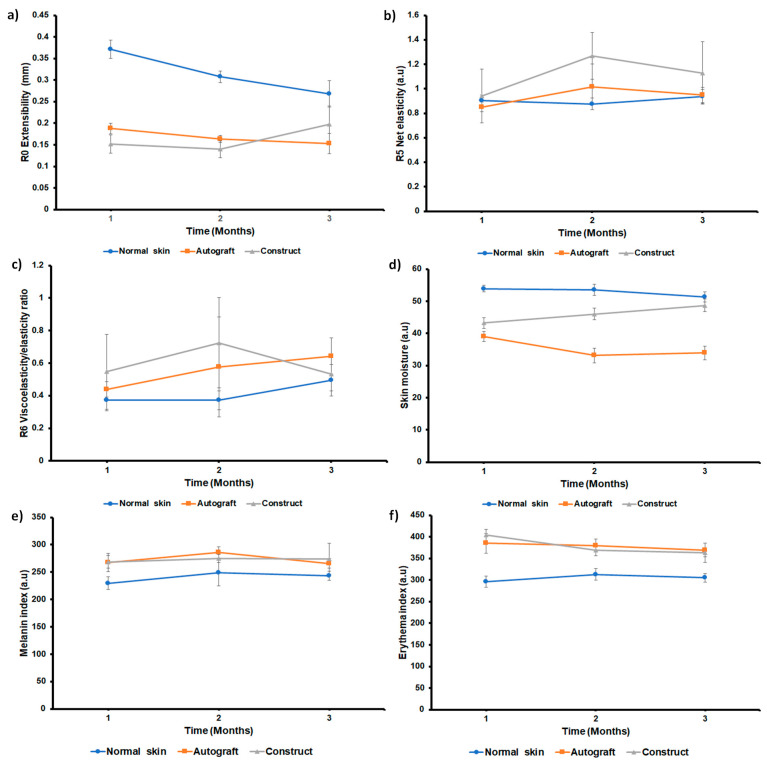
Biophysical properties of the skin. (**a**) R0, “extensibility” (**b**) R5 “net elasticity”, and (**c**) R6 “ratio of viscoelasticity to elasticity”. (**a**,**c**) the measurements were taken with a cutometer probe. (**d**) Skin moisture assessment by corneometer, (**e**) melanin index assessment by mexameter, and (**f**) erythema index assessment by mexameter. One, two, and three months after the injury, measurements were taken in the areas treated with the autograft and the construct. As a control, normal skin near the damaged region was also evaluated. Values were expressed as mean ± standard deviations from three independent measurements.

The biomechanical properties of the injured skin were determined at 1, 2, and 3 months after the burn, and normal skin near the damaged area was used as a control. Time-strain curves generated by the Cutometer showed that extensibility (R0), net elasticity (R5), and the ratio of viscoelasticity to elasticity (R6) changed throughout the follow-up period of time (Figure 10). The area treated with autograft and autologous construct showed greater firmness after 1 and 2 months than normal skin. However, three months later, the area treated with the autologous construct showed R0 values close to normal skin (Figure 10a). After three months, net elasticity values were similar between the autograft-treated area and the normal skin. At 2 and 3 months, the autologous construct-treated area showed higher R5 values than autograft and normal skin (Figure 10b). After three months of treatment, the autograft-treated region had a higher ratio of viscoelasticity-elasticity (R6) than the normal skin and construct-treated area (Figure 10c).

Skin moisture values were lower in the autograft-treated and construct-treated areas than in the normal skin. However, the construct-treated area showed an increase in skin moisture values in the function of time; after 3 months, the values were closer to normal skin, whereas the autograft-treated area showed lower values (Figure 10d). The areas treated with the autograft and the construct showed higher melanin values than normal skin at 1 and 2 months. However, at three months the values were similar to normal skin (Figure 10e). The erythema index was similar in areas treated with autograft or construct. Both areas show a decrease in the erythema index as a function of time. However, at 1, 2, and 3 months after injury, the erythema values in the treated regions were higher than in normal skin (Figure 10f).

## 4. Discussion

Extensive burns still represent a significant health problem in developing countries, mainly, because the lack of healthy skin to give autografts, patients with significant burn areas have to stay more time in bed to be treated with their own skin. Tissue engineering skin substitutes are available in first-world countries; however, in developing countries there are few tissue engineering substitutes available to treat their patients. In most of them, there are tissue banks in which pig skin is processed in order to be used as dressings since the donation culture is a problem in order to obtain human skin. Pig skin has been used as a dressing since the 1980s, but the dressing does not contribute to producing new tissue [4,25]. Previous work has shown that RPS is a good scaffold for the support of MSC which can differentiate into fibroblast and vascular tissue, immunoregulate the wound, release growth factors, and enhance ECM deposition which improves wound healing [6,7,14]. Here, it was shown that Fb and Kc can also be grown in RPS and could work as a scaffold to set the cells into the wound, the cells cultured on RPS showed high cell viability by Calcein and MTT (Figure 2), good cell attachment by SEM analysis (Figure 3), and the capability to release FGF (Figure 4).

The increase in multidrug-resistant bacteria in the world has generated the need for developing alternatives to the use of antibiotics, in response to this. The research on AgNPs has increased in the last few decades resulting in a good option to eradicate pathogenic bacteria and even biofilm formation [14]. Previous studies with AgNPs and clinical strains multiresistant Gram-negative and Gram-positive bacteria showed antimicrobial activity against the planktonic form, and moreover, antibiofilm activity was achieved against isolates from burned patients [6,7,14]. In skin wounds, sepsis is one of the biggest problems to achieve wound healing, in this regard, many wound dressings have been developed to avoid infection, one of them that is already in the market is ActicoatTM and it has shown good results to improve wound healing [13]. In the present research, RPS-AgNPs125 has been created, using RPS and AgNPs 125 ppm solution, to prevent the loss of water and bacterial infection and has been studied as a dressing that prevents bacterial infections without being in direct contact with the wound.

The negative effect of silver nanoparticles in the cell cycle, viability, and proliferation has been seen in other works, so caution should be taken in the concentration to which the cells are exposed [26,27]. However, in this work a relatively low concentration of AgNPs was used (125 ppm), this AgNPs’ concentration allows MSC to adhere to the RPS-AgNPs125 and remain alive; the micrographs show a good cell density without showing an accumulation of dead cells (Figure S2) on the scaffold surface (red cells). The use of MSC, Fb, and Kc plays an important role in wound healing, and they were used as in vitro models to analyze the possible cytotoxic effect of AgNPs, AgNPs reduce cell viability but at 125 ppm most of the cells can adhere to the scaffold and present adequate viability; however, AgNPs should be handled with caution [28,29]. The use of materials of biological origin like RPS, promotes a better interaction without leaving adverse effects on the damaged tissue, in addition, this type of material is eventually detached, which allows the incorporation of the cells naturally in the affected area [30]. One important result is that the incorporation of AgNPs to RPS allowed the closure of the wound in the animal model, showing that the AgNPs’ concentration applied here did not interfere with the wound healing process and also enhanced ECM deposition (Figure 5, Figure 6 and Figure S3 and S4). In a recent study, it was observed that when applying AgNPs (2–14 nm) suspended chronically at 1 mM in albino mice, no presence of silver was detected in internal organs, nor negative effects on the area affected by the burn. Additionally, the use of AgNPs showed less damage in the skin than silver sulfadiazine treatment, which is a classic treatment for burn injuries, these data are consistent with the results obtained in our experiments [31]. Another study showed that in rats with continuous administration of AgNPs for 4 weeks with 100 and 500 mg/kg/day, an accumulation of AgNPs in the blood, kidney, liver, or spleen was not found [29]; this study supports the data collected in the present research, in this case, it used low concentrations of AgNPs (125 ppm) in order to prevent bacterial infections but also to allow the attachment and proliferation of cells. In contrast, the silver sulfadiazine formulation used in previous studies shows zero biocompatibility with fibroblasts and biodistribution to organs such as the brain [31,32,33], due to the high concentration of silver and the components of the formulation, which corroborate that incorporation of silver nanoparticles should be carried out at low and controlled concentrations (null or sustained release) and in materials that guarantee biocompatibility with the host.

Here, the RPS-AgNPs125 was used with a low concentration of AgNPs and the objective was to prevent microorganism infections; for the clinical phase, the RPS-AgNPs125 was applied over the autologous cellular construct (RPS seeded with Fb and Kc) in the case of study and it never was in direct contact with the wound.

The autologous cellular (RPS-FB-Kc) construct applied to the burn patient showed the same recovery as skin autograft, which indicates that this tissue engineering skin substitute is a good option to grow skin cells and help to cover the wounds in patients with extensive burns. Skin properties such as elasticity, moisture, erythema index, and melanin index by non-invasive methods have been used for diagnostic purposes to evaluate the clinical performance of the autologous cellular (RPS-FB-Kc) construct covered by the RPS-AgNPs125 antibacterial cover as an integral treatment for deep second-degree burns [15]. This study evaluated the skin properties after treatment by comparing normal skin near the burn-damaged area with the areas treated with skin autograft and engineered autologous cellular construct. Parameters related to biomechanical properties such as extensibility, net elasticity, and the ratio of viscoelasticity to elasticity were similar in the areas treated with the construct and with the skin autograft. However, the biomechanical properties of the treated damaged areas were different from those of uninjured skin. The construct-treated area showed better skin moisture than the autograft-treated area, and the melanin and erythema index was higher than normal skin. These results suggest that the construct substitute is close to the parameters obtained with skin autografts, which supports the idea of them being used simultaneously while the patient is treated with the gold standard to reduce hospitalization time (Figure 10).

Finally, it is important to mention that the present study has some limitations that should be addressed. Firstly, it was conducted on a single patient, and in order to strengthen the reliability of the data obtained, it is crucial to recruit a larger number of patients. This will help to ensure the generalizability and applicability of the findings to a broader population. Additionally, although tissue engineering holds promises as an effective approach for treating patients with extensive burns, the time required to develop the autologous cellular constructs is long. Therefore, further optimization of the process to obtain the appropriate number of cells to develop the autologous cellular construct is necessary to expedite the production of these constructs without compromising their quality and efficacy. Furthermore, in the mouse model, no silver deposition was observed in the skin or organs. However, considering that the RPS-AgNPs125 was not in direct contact with the skin of the patient, it is essential to investigate the distribution and potential accumulation of silver in other tissues, such as blood and skin, within the patient’s body. Examining these aspects will provide a comprehensive understanding of the systemic effects and safety profile of the nanocomposite in a clinical setting. Addressing these limitations will contribute to the overall validity and applicability of the present study’s findings and will provide valuable insights for the further development and application of the RPS-AgNPs nanocomposite and RPS-Fb-Kc autologous construct in wound healing and tissue engineering approaches.

## 5. Conclusions

Radiosterilized pig skin guarantees good biocompatibility with Fb, Kc, and MSC and it can be used as a scaffold to develop cellular constructs to treat chronic wounds such as extensive burns, in addition, its easy accessibility and low cost allow a more affordable therapy for the general population. The antibacterial cover generated with a low concentration of AgNPs, that is RPS-AgNPs125, improved wound healing in an animal model and can be used as an antibacterial dressing in order to prevent infection of the wound. The autologous cellular RPS-FB-Kc construct and the RPS-AgNPs125 antibacterial cover combined restored a second-degree burn in a patient with similar results to skin autografts.

## Figures and Tables

**Table 1:** Antibiofilm properties of the nanomaterial against *S. aureus*.

RPS-AgNPs Cover	Log Reduction * (±SD)
RPS-AgNPs1000	3.27 ± 0.12
RPS-AgNPs500	2.28 ± 0.23
RPS-AgNPs350	2.07 ± 0.20
RPS-AgNPs250	1.9 ± 0.18
RPS-AgNPs125	1.74 ± 0.24

* Biofilm Log reduction in comparison to RPS with no AgNPs (negative control).

**Table 2:** Silver concentration in organs. N.D. = not detected.

	RPS-AgNPs125	AgNPs Suspension at 1000 ppm
	Silver Concentration (ppm/g Tissue)	
Heart	N.D.	N.D.
Liver	N.D.	N.D.
Spleen	N.D.	N.D.
Kidney	N.D.	N.D.

## Data Availability

Data are available upon request from the corresponding author.

## Supplementary Materials

The following supporting information can be downloaded at: https://www.mdpi.com/article/10.3390/pharmaceutics15082105/s1, Figure S1. Procedure for the implantation of antibacterial cover; RPS-AgNPs125. Figure S2. The RPS and RPS-AgNPs125 allow cell attachment and viability of MSC. Figure S3. Hematoxylin & eosin analysis of skin tissue after treatment. Figure S4. Masson stain analysis of treatments. Figure S5. Development of the construct and the RPS-AgNPs125 cover before implantation.
