# Supplementary material for: Radiosterilized Pig Skin, Silver Nanoparticles and Skin Cells as an Integral Dressing Treatment for Burns: Development, Pre-Clinical and Clinical Pilot Study
Source: Pharmaceutics. 2023 Aug 9;15(8):2105. doi: 10.3390/pharmaceutics15082105 (PMC10458621; doi:10.3390/pharmaceutics15082105)
Supplement: Supplementary file 1 [file pharmaceutics-15-02105-s001.zip › pharmaceutics-2520032-supplementary.pdf]

Supplementary material

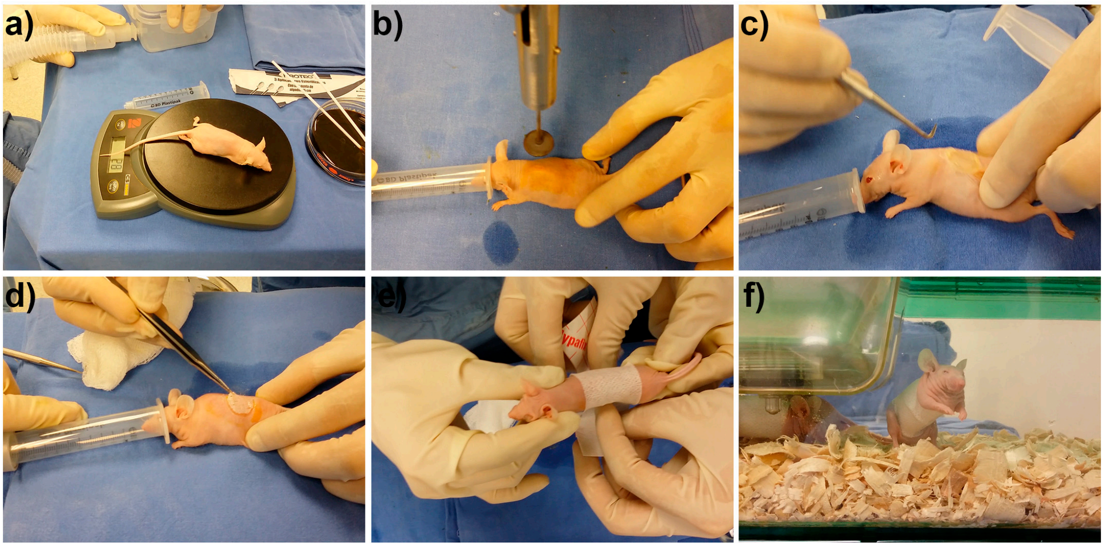

Figure S1. Procedure for the implantation of antibacterial cover; RPS-AgNPs125. a) initial mouse weighing after anesthesia with isoflurane 6%; b) second-degree deep burn (2 cm^2) induction on the back of the mouse using a 366 g apparatus with a circular cupper tip at 225 ^{0}C per 5 seconds in contact with the mouse back; c) debridement and washing of the burned area with PBS; d) application of treatments (gauze, RPS, RPS-AgNPs125 or AgNPs in 1000 ppm suspension); e) complete wound dressing with Hypafix® and f) analgesia application and mouse consciousness recovery.

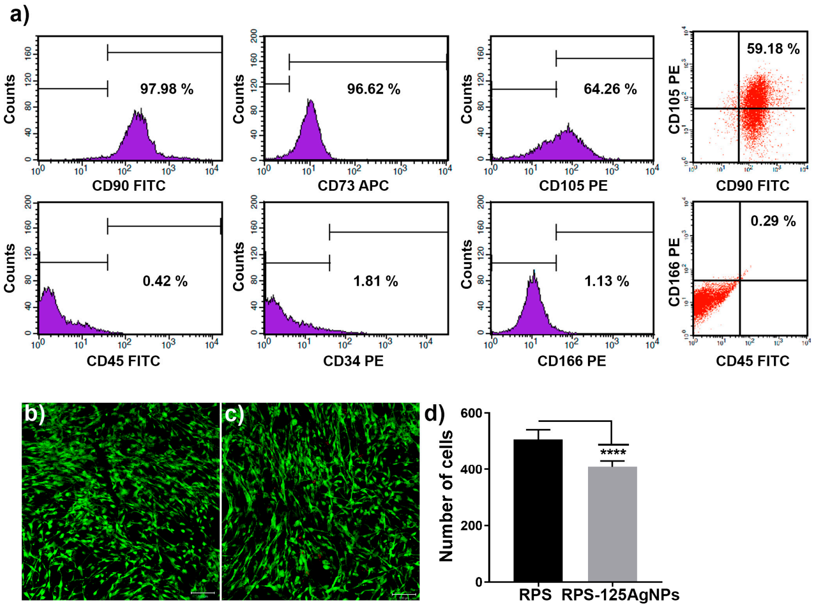

Figure S2. The RPS and RPS-AgNPs125 allow cell attachment and viability of MSC. a) Data correspond to the percentage of MSC labeled with primary monoclonal antibodies conjugated to a fluorochrome and analyzed by flow citometry. The upper panel graphs display MSC expression for CD90-FITC, CD73-APC and CD105-PE. Lower panel graphs show expression for hematopoietic stem cell markers CD45-FITC, CD34-PE, and CD166-PE. Representative live/dead assay photograph showing MSC seeded and culture for 48 h on b) RPS and c) RPS-125AgNPs; viable cells are stained with calcein (green) and dead cells with ethidium homodimer (red). d) The graph represents the number of cells present on RPS and RPS-125AgNPs after 48 h of culture. ****p< 0.0001 unpaired Student´s t analysis.

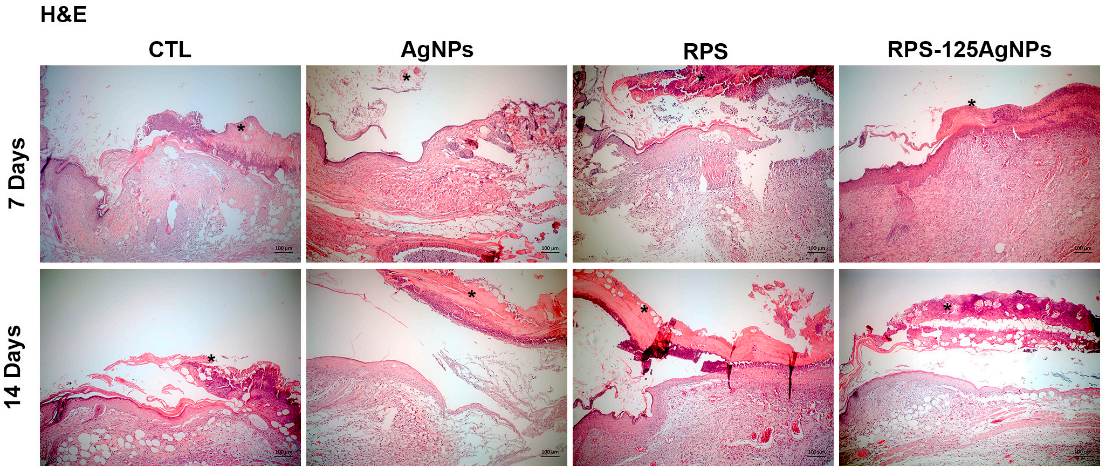

Figure S3. Hematoxylin & eosin analysis of skin tissue after treatment. Representative photographs of wound area tissue stained with H&E, at seven and fourteen days after the different treatments. The images were taken at the edges of the lesion, where the epithelium begins to re-epithelize the burn area. Scale bars correspond to 100 μm.

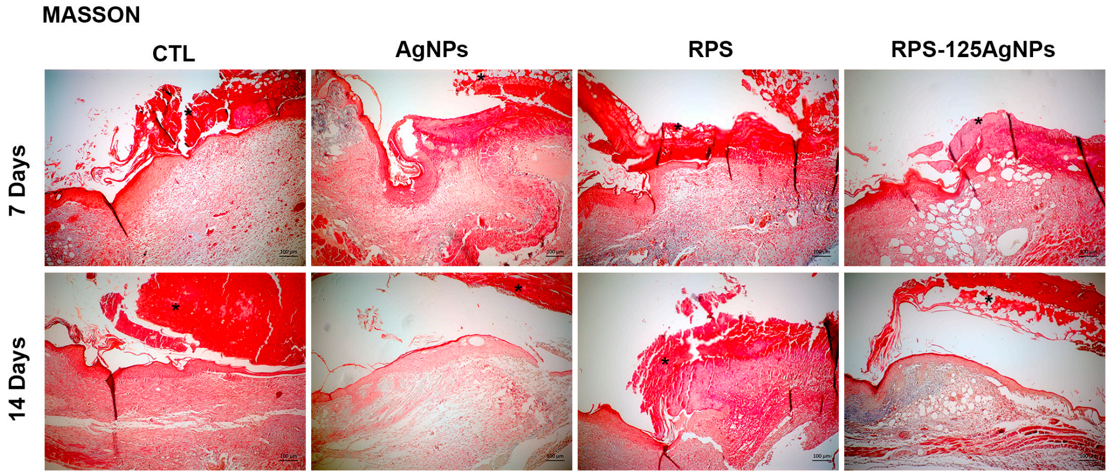

Figure S4. Masson stain analysis of treatments. The image shows photographs of histological sections at day seven and fourteen after treatment, stained with Masson. In red it is possible to observe the epidermis and the crust (*), only in the treatment with RPS and RPS-AgNPs125 at fourteen days it is observed a blue stain (indicating collagen deposition) under the new developed epidermis. Scale bars correspond to 100 μm.

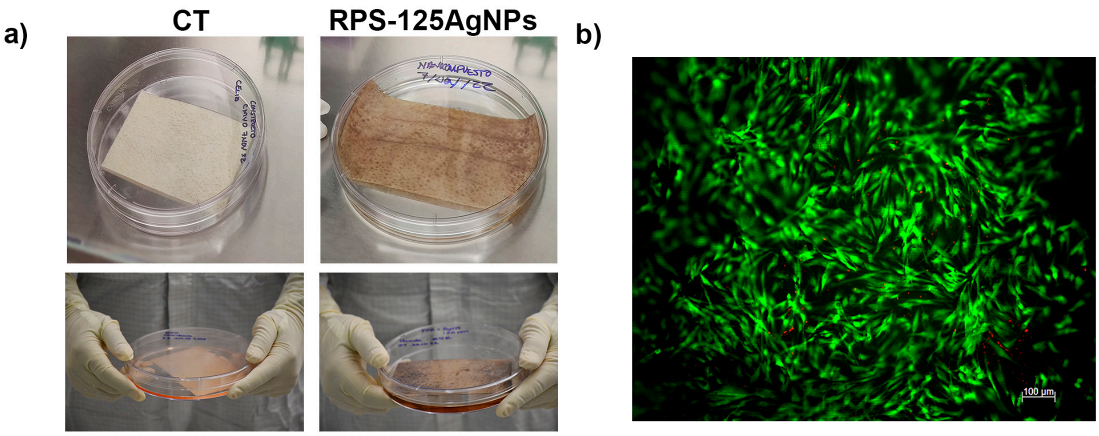

Figure S5. Development of the construct and the RPS-AgNPs125 cover before implantation. a) The image at the left upper corner shows the autologous cellular construct, and on the right upper corner, the RPS-AgNPs125 is shown. b) Calcein/Eth-D1 cell viability assay on the construct at the time of implantation.
